# Potential Bidirectional Relationship Between Periodontitis and Alzheimer’s Disease

**DOI:** 10.3389/fphys.2020.00683

**Published:** 2020-07-03

**Authors:** Daniela Liccardo, Federica Marzano, Federica Carraturo, Marco Guida, Grazia Daniela Femminella, Leonardo Bencivenga, Jacopo Agrimi, Armida Addonizio, Imma Melino, Alessandra Valletta, Carlo Rengo, Nicola Ferrara, Giuseppe Rengo, Alessandro Cannavo

**Affiliations:** ^1^Department of Translational Medical Sciences, University of Naples Federico II, Naples, Italy; ^2^Center for Translational Medicine, Temple University, Philadelphia, PA, United States; ^3^Department of Advanced Biomedical Sciences, University of Naples Federico II, Naples, Italy; ^4^Department of Biology, University of Naples Federico II, Naples, Italy; ^5^Division of Cardiology, Johns Hopkins University, Baltimore, MD, United States; ^6^Department of Neurosciences, Reproductive and Odontostomatological Sciences, University of Naples Federico II, Naples, Italy; ^7^Department of Prosthodontics and Dental Materials, School of Dental Medicine, University of Siena, Siena, Italy; ^8^Istituti Clinici Scientifici ICS Maugeri - S.p.A.-Istituti di Ricovero e Cura a Carattere Scientifico (IRCCS) Istituto Scientifico di Telese Terme, Telese, Italy; ^9^Task Force on Microbiome Studies, University of Naples Federico II, Naples, Italy

**Keywords:** Alzheimer’s disease, periodontitis, dysbiosis, neurodegeneration, dementia

## Abstract

Alzheimer’s disease (AD) is the most prevalent form of dementia in the elderly population, representing a global public health priority. Despite a large improvement in understanding the pathogenesis of AD, the etiology of this disorder remains still unclear, and no current treatment is able to prevent, slow, or stop its progression. Thus, there is a keen interest in the identification and modification of the risk factors and novel molecular mechanisms associated with the development and progression of AD. In this context, it is worth noting that several findings support the existence of a direct link between neuronal and non-neuronal inflammation/infection and AD progression. Importantly, recent studies are now supporting the existence of a direct relationship between periodontitis, a chronic inflammatory oral disease, and AD. The mechanisms underlying the association remain to be fully elucidated, however, it is generally accepted, although not confirmed, that oral pathogens can penetrate the bloodstream, inducing a low-grade systemic inflammation that negatively affects brain function. Indeed, a recent report demonstrated that oral pathogens and their toxic proteins infect the brain of AD patients. For instance, when AD progresses from the early to the more advanced stages, patients could no longer be able to adequately adhere to proper oral hygiene practices, thus leading to oral dysbiosis that, in turn, fuels infection, such as periodontitis. Therefore, in this review, we will provide an update on the emerging (preclinical and clinical) evidence that supports the relationship existing between periodontitis and AD. More in detail, we will discuss data attesting that periodontitis and AD share common risk factors and a similar hyper-inflammatory phenotype.

## Introduction

Alzheimer’s disease (AD) is a neurodegenerative disorder affecting millions of people worldwide, with a frequency that is rapidly rising as the life expectancy increases and the world population becomes older (Brookmeyer et al., 2002, 2007; Sosa-Ortiz et al., 2012; Alzheimer’s Association., 2016). Importantly, AD is characterized by neuronal loss with a slow and progressive decline in memory, language, and other cognitive skills, leading to the final stage of the disease, which is ultimately fatal (Alzheimer’s Association 2016).

Despite decades of intense investigation, how degenerative neurodisorders, such as AD, develop remains unclear. Aggregates (plaques) of the amyloid-β peptide (AβP), as well as neurofibrillary tangles of the hyperphosphorylated protein, tau, are among the most sought-after therapeutic targets for AD (Braak and Braak, 1995; Long and Holtzman, 2019). However, many clinical trials investigating the effects of anti-amyloid drugs failed to demonstrate improvement in patients’ cognitive performance and in countering the primary adverse events (Pinheiro and Faustino, 2019). Hence, there is an increasing interest in identifying new strategies to prevent and/or treat AD. For instance, several modifiable risk factors have been considered so far, such as physical inactivity, mood disorders, hypertension, diabetes mellitus, and obesity (Mayer et al., 2018). Moreover, many reports are now supporting the role of inflammation as a significant pathological driver of AD development and cognitive decline, with evidence that communication between the brain and peripheral immune systems also exists (Goldeck et al., 2016; Cao and Zheng, 2018; Alexandraki et al., 2019; Long and Holtzman, 2019; Tejera et al., 2019). In this sense, multiple studies have raised that an infectious hypothesis might underlie the pathogenesis of AD (Long and Holtzman, 2019). For instance, several studies have demonstrated the presence of herpesvirus (HSV) within the amyloid plaques and in the brains of AD patients (Jamieson et al., 1991; Jamieson et al., 1992; Wozniak et al., 2009; Carbone et al., 2014). In line with these data, HSV-1 particles can directly induce the fibrillization of Aβ42 *in vitro* (Ezzat et al., 2019). Moreover, two retrospective cohort studies demonstrated that HSV infection significantly increased the risk of developing all-cause dementia. Of note is that this risk was almost eliminated in patients treated with antiherpetics (Chen et al., 2018; Tzeng et al., 2018; Long and Holtzman, 2019).

Further to these viral effects on AD development, the research in the field has focused its attention on periodontitis, a chronic oral inflammatory condition, and its potential bidirectional link with AD (Kim and Amar, 2006; Kamer et al., 2008; Chen et al., 2017; Marchini et al., 2019; Long and Holtzman, 2019). Importantly, people with periodontitis have an increased risk of developing AD (Chen et al., 2017), and those with AD or dementia have impaired oral health, as a result of cognitive decline, and are more prone to develop chronic oral diseases, such as periodontitis, tooth loss, and mucosal lesions (Tada et al., 2006; Gonsalves et al., 2008; Noble J.M. et al., 2013; Maldonado et al., 2018). Mechanistically, periodontal pathogens not only invade the oral cavity but can also percolate through the epithelium of the periodontal pocket. From here, they can enter the bloodstream, where they can induce the release of several endotoxins and exotoxins, thus fueling infection in different compartments, including the brain (Nazir, 2017; Sudhakara et al., 2018; Bui et al., 2019; Dominy et al., 2019; Liccardo et al., 2019).

Thus, this review aimed to provide the readers with an update on the most recent findings that support the existence of a relationship between periodontitis and AD, with particular emphasis on the common risk factors, phenotype, and bidirectionality.

## Pathophysiology of Alzheimer’s Disease: β-Amyloid, Tau, and ApoE

AD is generally classified into two forms: the inherited and the sporadic one (Bekris et al., 2010; Dorszewska et al., 2016). Although there are differences in terms of the triggering factors and the proportion of the affected population, the underlying neuropathology of both conditions remains similar: with patients progressing from normal to mild cognitive impairment (MCI), followed by increasing dementia severity, eventually leading to the final stage of the disease that is ultimately fatal (Donev et al., 2009; Sperling et al., 2011; Scheltens et al., 2016; Davis et al., 2018). At a molecular level, both the sporadic and the inherited forms are characterized by the same diagnostic hallmarks such as AβP plaques and neurofibrillary tangles (Braak and Braak, 1995; Long and Holtzman, 2019).

### AβP Plaques

**FIGURE 1:**
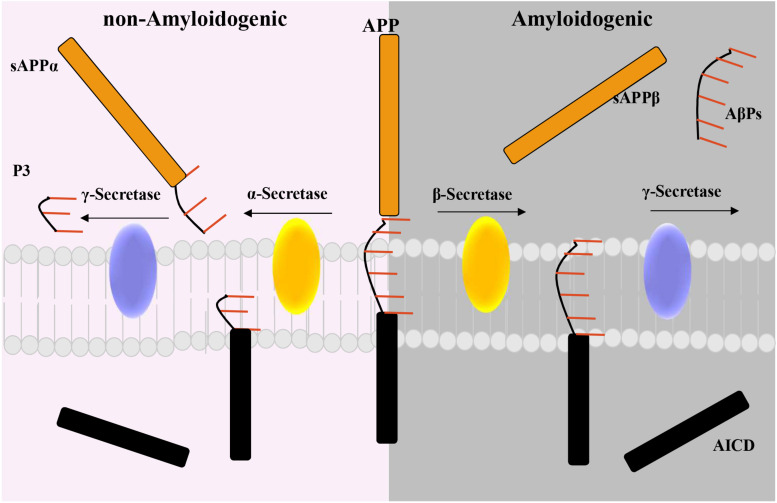
Diagram of the non-amyloidogenic and the amyloidogenic proteolytic pathway for the amyloid precursor protein (APP). Non-amyloidogenic pathway: α-secretase cleaves the transmembrane protein APP to release the soluble APP fragment, sAPPα. The APP C-terminal fragment is then processed by γ-secretase to release an intracellular domain (AICD) and the P3 fragment. Amyloidogenic pathway: β-secretase processes APP to generate the soluble fragment, sAPP-β, then cleaved γ-secretase Aβ peptides (AβPs), and the AICD.

In 1907, Alois Alzheimer, a German neurologist, reported the presence of a not well-identified substance in the cortex associated with a progressive behavioral and cognitive disorder (Alzheimer et al., 1995; O’Brien and Wong, 2011). Almost 80 years later, Glenner and Wong (1984) demonstrated that this substance was constituted by a ∼4 kDa peptide called AβP. AβP is a fragment derived from the proteolytic cleavage of the amyloid precursor protein (APP). APP is a transmembrane protein with a large ectodomain, a C-terminal (CT) membrane-bound domain and short intracellular domain (AICD) (Passer et al., 2000; Serpell, 2000; Gu et al., 2001; Weidemann et al., 2002; Kakuda et al., 2006; Walsh and Selkoe, 2007). Importantly, two main proteolytic pathways have been described for APP: the nonamyloidogenic and the amyloidogenic (Andrew et al., 2016; Figure 1). In the non-amyloidogenic pathway, α-secretase ADAM10 cleaves APP within the Aβ domain, generating a soluble proteolytic fragment, termed sAPPα, and a membrane-bound CT fragment (CTFα). Importantly, CTFα is subsequently processed by another proteolytic process that involved γ-secretases to generate p3 and the AICD. Conversely, in the amyloidogenic pathway, β-secretase 1, also known as β-site APP cleaving enzyme 1 (BACE1), and presenilin-containing γ-secretase (PS/γ-secretase) multi-subunit complex are involved in the generation of AβP (De Strooper et al., 1998; Struhl and Greenwald, 1999; Wolfe et al., 1999; Gu et al., 2001; De Strooper et al., 2012; Ben Halima et al., 2016; Andrew et al., 2016). More in detail, BACE1 cleaves APP, liberating a sAPPβ fragment and a 99-amino acid remaining CTF (CTFβ) (De Strooper et al., 2012; Andrew et al., 2016; Ben Halima et al., 2016). Then, the CTFβ is processed at the ε-site by PS/γ-secretase, thereby releasing the AICD (Andrew et al., 2016; Ben Halima et al., 2016). The AICD, either produced by α- or β-secretase, translocates into the nuclei of neurons. Here, it acts as a regulator of gene expression, including that of the Aβ-degrading neprilysin, or is degraded into the cytosol (Belyaev et al., 2010; Grimm et al., 2015; Multhaup et al., 2015). Importantly, different Aβ forms are generated by PS/γ-secretase cleavages at the ζ and γ sites that trim the transmembrane domain of CTFβ to liberate several forms of AβPs of variable lengths [from 38 (Aβ38) to 42 (Aβ42) amino acids] (De Strooper et al., 2012). In this regard, Aβ40 is the major product generated, along with minor amounts of Aβ38 and Aβ42 (De Strooper et al., 2012). However, besides these Aβ forms, it has been reported that, in this process, tiny amounts of Aβ37 and Aβ43 are also generated (De Strooper et al., 2012).

Importantly, although AβP’s function is still debated and uncertain, these products are generated throughout life and appear to be normally stimulated by synaptic activity (Pearson and Peers, 2006). Conversely, dysregulation of the trimming process of APP can lead to a substantial increase in the levels of the insoluble Aβ42 isoform. This isoform is more prone to form oligomers, which correlate with synaptic dysfunction (Hayden and Teplow, 2013), protofibrils, or fibrils. Importantly, Aβ42 oligomers represent the most soluble and potent toxic conformers of AD (Haass and Selkoe, 2007), and their presence correlates with the severity of the disease (McLean et al., 1999). However, as recently suggested by Gulisano et al. (2018), Aβ42 oligomers are crucial both in physiological and pathological conditions. Indeed, only when present in excessive concentrations or for a prolonged time do these Aβ isoforms can negatively affect long-term potentiation (LTP) and memory. Conversely, low-dose administration positively affects synaptic plasticity and memory.

Thus, all the described processes, in a multitude, generate amyloid plaques resulting toxic to neurons and participating in synaptic destruction during the early stages of AD (Andrew et al., 2016). However, it is worth stressing that several studies are now supporting the idea that amyloid plaques are not the major toxic AβP entity, and amyloid plaques are not a direct indicator of AβP-induced brain damage in AD. For instance, the Arctic APP mutation (E693G) (Nilsberth et al., 2001) leads to enhanced Aβ protofibril formation and AD dementia. Still, no amyloid is visible on positron emission tomography (PET) imaging through the ^11^C-labeled Pittsburgh Compound B (PiB) ligand (Schöll et al., 2012). Similarly, the Osaka mutation (E693Δ) in APP causes the aggregation of AβP with little amyloid accumulation on PiB-PET (Shimada et al., 2011). Similarly, transgenic mice carrying the Osaka mutation do not show, by immunohistochemistry, amyloid deposits (Tomiyama et al., 2010). Thus, several therapeutic strategies targeting Aβ have been tested in the last decades, such as secretase inhibitors, AβP aggregation inhibitors, and Aβ immunotherapy (Pinheiro and Faustino, 2019). However, almost all of these strategies have been discontinued, either because of side effects or the lack of sizable therapeutic effects. Nevertheless, the failure of past clinical trials targeting Aβ does not mean that Aβ is a wrong target. Indeed, the current common concern is that AD patients must be treated at an earlier stage, i.e., right when the pathological “amyloid” cascade likely begins.

### Tau Protein

The protein tau has been identified and purified in 1970 (Weingarten et al., 1975; Cleveland et al., 1977) as a microtubule-interacting protein that stabilizes the neuronal cytoskeleton. The tau protein structure is composed of four main regions: an acidic N-terminal (NT); a proline-rich region responsible for the binding to microtubules; four repeat domains (R1–4), also called microtubule-binding domains (MBDs) (Drewes et al., 1995; Sengupta et al., 1998; Gendron and Petrucelli, 2009); and a C-terminal (CT) region. Importantly, tau activity can be modulated by a wealth of posttranslational modifications (PTMs), such as acetylation, glycosylation, glycation, methylation, truncation, nitration, ubiquitination, and phosphorylation (Almansoub et al., 2019). However, phosphorylation is the most commonly described and investigated since it is centrally involved in the formation of pathologic aggregates. Indeed, the aggregation of tau has been correlated to a broad spectrum of neurological diseases, including AD, known as “tauopathies” (Congdon and Sigurdsson, 2018; Almansoub et al., 2019). This PTM is physiologically regulated by the balance between tau kinases and phosphatase activities (Martin et al., 2013). Importantly, among 85 phosphorylation sites, about 45 of these are phosphorylated in AD brains (Noble W. et al., 2013). More in detail, the early phosphorylation events, at specific serine residues such as Ser199, Ser202/205, and Ser262, can disrupt the association of tau with microtubules. This event, in turn, can lead to alterations in tau-dependent cellular functions with dysregulated axonal growth and vesicle and organelle transport (Gendron and Petrucelli, 2009; LaPointe et al., 2009; Congdon and Sigurdsson, 2018). Otherwise, phosphorylation at other serine residues, such as Ser396, has been suggested as a prominent subsequent event that correlates with the progression of AD (Congdon and Sigurdsson, 2018).

Like phosphorylation, tau acetylation may arise from multiple mechanisms, and the dysregulation of this process can chiefly contribute to neurodegeneration. For instance, acetylation appears to prevent the binding of ubiquitin and then tau turnover (Min et al., 2010). This event can prompt a rise in cytosolic tau levels that makes the protein prone to aggregation (Congdon and Sigurdsson, 2018). Finally, unlike other tau posttranslational modifications, *O*-GlcNAcylation seems to be protective against tau-induced pathology. Indeed, in the AD brain, the levels of *O*-GlcNAcylated tau are reduced when compared to those in healthy subjects (Liu et al., 2009). Thus, targeting these posttranslational modifications may offer new avenues to prevent tau aggregation, restoring the normal function of the protein.

### Apolipoprotein E

Apolipoprotein E (ApoE) is a primary cholesterol carrier highly expressed in astrocytes and, to a lesser extent, in the microglia which mediates both the transport and delivery of lipids from a cell type to another (Mahley and Rall, 2000). In humans, three different alleles (ε2, ε3, and ε4) give rise to three different isoforms of ApoE, which differ in amino acids in positions 112 and 158: ApoE2 (Cys112 and Cys158), ApoE3 (Cys112 and Arg158), and ApoE4 (Arg112 and Arg158) (Mahley and Rall, 2000; Liu et al., 2013). Importantly, the single amino acid difference in the ApoE protein influences its ability to bind lipids, receptors, and also Aβ(REFF). Indeed, several studies have demonstrated that ApoE has a crucial role in Aβ aggregation and clearance influencing senile plaque formation and AD development (Ellis et al., 1996; Liu et al., 2013). In this context, several reports, including clinical, epidemiological, and genetic studies, have demonstrated an association between ApoE genotypes and AD. For instance, genome-wide association studies (GWAS) have confirmed that the ε4 allele of ApoE is one of the strongest genetic risk factors for AD (REFF). Indeed, the ε4 allele is significantly enriched in AD patients (Corder et al., 1993) and is associated with an increased Aβ plaque load in the brain (Schmechel et al., 1993), a higher brain atrophy (Agosta et al., 2009), and an earlier onset and accelerated progression of the disease (Shi et al., 2017).

Moreover, it has been shown that Aβ deposition and aggregation to form senile plaques are a phenomena predominantly observed in ApoE ε4 allele carriers compared with non-carriers (Schmechel et al., 1993; Polvikoski et al., 1995; Kok et al., 2009). In addition, ApoE ε4 carriers present lower Aβ42 levels in cerebrospinal fluids (CSFs) and higher PiB-positive imaging (Prince et al., 2004; Head et al., 2012).

## Neuroinflammation in the Pathogenesis of Alzheimer’s Disease

In addition to the two classic diagnostic hallmarks of AD, Aβ plaques and neurofibrillary tangles, the brain of patients with AD exhibits evidence of a sustained inflammatory response (Mandrekar and Landreth, 2010; Femminella et al., 2018, 2019). In the acute phase, inflammation in the brain represents an established defense against infections, toxins, and injury. However, a disruption in the equilibrium between the pro- and anti-inflammatory mediators results in a chronic inflammatory condition of the brain which is identified as a neuroinflammation (Mandrekar and Landreth, 2010). Importantly, this process is currently attributed to the accumulation of reactive microglia and astrocytes that, in AD, appears to be localized to amyloid deposits (Alzheimer et al., 1995; Bornemann et al., 2001; Stalder et al., 2001; Mandrekar and Landreth, 2010). Microglia are the resident phagocytes of the central nervous system that are activated in response to AβP accumulation, change their morphology to ameboid cells, migrate to the plaques, and release inflammatory mediators, starting the phagocytosis of the plaques (Kettenmann et al., 2011; Du et al., 2017; Wolf et al., 2017). However, while in the acute phase, the activation of microglia is neuroprotective; in chronic phase, it exacerbates neuroinflammation with consequent neurodegeneration (Solito and Sastre, 2012; Heppner et al., 2015; Zuroff et al., 2017; Kinney et al., 2018). Emerging evidences have demonstrated that astrocyte-mediated neuroinflammation is also involved in the pathogenesis of neurodegenerative diseases, including AD (Verkhratsky et al., 2010; Colombo and Farina, 2016). Astrocytes are specialized glial cells involved in the production of neurotrophic factors and in the maintenance of the blood brain–barrier (BBB), which protect the central nervous system (CNS) from harmful molecules and cells (including pathogens) (Sofroniew and Vinters, 2010). In response to brain insults, these cells become activated, a process known as reactive astrogliosis, and they release reactive oxygen species (ROS), nitric oxide (NO), and pro-inflammatory molecules, including interleukins (ILs) and tumor necrosis factor (TNF) (Phillips et al., 2014; Neal and Richardson, 2018). Although initially this process is aimed at removing noxious stimuli, prolonged astrocyte activation causes detrimental effects, leading to neuronal dysfunction and cell loss (Steardo et al., 2015). Reactive astrogliosis is a hallmark of AD and is responsible for the exacerbation of AβP-induced neurotoxicity and increased tau phosphorylation (Garwood et al., 2011; Osborn et al., 2016).

The involvement of neuroinflammation in the pathogenesis of AD has been supported by observational and epidemiological studies demonstrating that chronic use of nonsteroidal anti-inflammatory drugs (NSAIDs) can exert beneficial roles in reducing the risk of AD (Ali et al., 2019). Moreover, mutations in the genes encoding for immune receptors, including triggering receptor expressed on myeloid cells 2 (TREM2) and myeloid cell surface antigen CD33, have been associated with an elevated risk of developing AD (Griciuc et al., 2013; Gratuze et al., 2018). TREM2 is a transmembrane immune receptor expressed on the surface microglia, and in AD, it is involved in the clearance of Aβ plaques (Boche et al., 2013; Jevtic et al., 2017; Lagarde et al., 2018). For this reason, an alteration in TREM2 function is reported as harmful and correlates with AD development. For instance, Wang and coworkers have demonstrated that TREM2 deficiency resulted in an increased AβP accumulation in the brain with reduced clustering of the microglia around the plaques (Wang et al., 2015). Importantly, the most common TREM2 mutation is the arginine 47 histidine (R47H) variant, which appears to be associated with a reduced microglial uptake of Aβ and an increased risk of AD development (Guerreiro et al., 2013; Jonsson et al., 2013; Tanzi, 2015; Hansen et al., 2018). In this regard, Cheng-Hathaway et al. (2018) demonstrated that AD mice heterozygous for the TREM2 R47H presented reduced immune cells and enhanced neuritic dystrophy around Aβ plaques. Importantly, other TREM2 variants have also been studied for their association with the risk of AD, including R62H (Huang et al., 2004; Guerreiro et al., 2013; Jonsson et al., 2013; Jin S.C. et al., 2014; Roussos et al., 2015; Ghani et al., 2016; Song et al., 2017; Sims et al., 2017). More in detail, Kleinberger et al. demonstrated, in human macrophages *in vitro*, that the TREM2 R62H variant led to an impairment of the phagocytic functions of TREM2 with a reduced uptake of Aβ-LDL complexes compared to wild-type control cells (Kleinberger et al., 2014; Yeh et al., 2016). Of note is that numerous recent findings suggest a link between tau protein aggregation and TREM2 dysfunction. For instance, in the CSF of AD patients, the levels of soluble TREM2 correlate with the amount of total and phosphorylated tau, but not with those of Aβ42 (Piccio et al., 2016). Importantly, either soluble TREM2 or the phosphorylated tau levels in the CSF are related to the cognitive decline and clinical progression of AD (de Leon et al., 2004; Buerger et al., 2006; Andersson et al., 2008; Ewers et al., 2019). Contrary to the protective role of TREM2, CD33 induces a negative response in AD because this receptor inhibits phagocytosis, thus reducing microglial uptake and clearance of Aβ (Griciuc et al., 2013). There is also evidence for the existence of a potential crosstalk between CD33 and TREM2. More in detail, Griciuc and coworkers have demonstrated, in a murine model of AD, that loss of CD33 resulted in a decreased Aβ pathology and improved cognition (Griciuc et al., 2019). However, these effects were significantly abrogated by additional TREM2 knockout (Griciuc et al., 2019). Conversely, TREM2 knockout mice presented increased Aβ pathology and exacerbated neurodegeneration, which was not rescued by additional knockout of CD33. Thus, the authors concluded that TREM2 acts downstream of CD33.

Importantly, an association between TREM2 and ApoE has also been discussed. For instance, Jendresen et al. (2017) have demonstrated that human ApoE protein contains a binding site for TREM2 (amino acids 130–149), and this binding is isoform-dependent. In line with this report, Atagi et al. (2015) showed that ApoE can increase the phagocytosis of apoptotic neurons *via* TREM2 binding.

Importantly, ApoE activity has also been associated with microglia function. Indeed, LaDu and colleagues have demonstrated that glial cells cultured from ApoE knockout (KO) mice show an increased production of pro-inflammatory markers in response to treatment with Aβ (LaDu et al., 2001). In line with these data, in 2003, Lynch et al. (2003) have demonstrated that intravenous administration of lipopolysaccharide (LPS) in animals expressing the ε4 allele resulted in a more significant systemic and brain inflammation compared with their ε3 allele counterparts. Analogously, in a tauopathy murine model, ApoE knockdown markedly reduced the activation of microglia and astrocytes (Shi et al., 2017). This evidence supports the role of ApoE in neurodegenerative disorders. In the same vein, in one report, Rodriguez and colleagues demonstrated a direct relationship between ApoE, neuroinflammation, and AD (Rodriguez et al., 2014). Indeed, in the cortex of transgenic mice expressing five familial AD mutations (FAD), these authors found that the ApoE genotype can influence both Aβ deposition and Aβ-induced glial activation. Consistent with this notion, NSAIDs have been shown to reduce AD risk only in ε4 allele carriers, further supporting the role of the *ApoE* genotype in AD progression and development (Szekely et al., 2008).

Finally, in addition to these mechanisms, chronic complement activation has been linked to neuroinflammation and AD (Fischer et al., 1995; Fischer and Popa-Wagner, 1996). In particular, recent pieces of evidence from GWAS have identified complement component receptor (CR1), which binds complement proteins C3b and C4b, as a risk factor for AD (Lambert et al., 2009). In line with these data, Brouwers and coworkers found four single-nucleotide polymorphisms (SNPs) in the CR1 locus that were associated with elevated levels of Aβ in the CSF of patients with AD (Brouwers et al., 2012). Furthermore, intragenic duplication of low copy repeats (LCR) within the CR1 gene appears to be associated with an increased risk of late-onset AD (Kucukkilic et al., 2018).

## Oral Dysbiosis, Inflammation, and Periodontitis

The microbiome plays a crucial role in human physiology influencing nutrition, immunity, organ development, and function (Sudhakara et al., 2018). In the last decades, the observation that several chronic diseases of the gastrointestinal tract and mouth are associated with the perturbation of microbiome (dysbiosis) has achieved growing attention from scientists. Thus, several studies have been designed to evaluate the potential association between dysbiosis and systemic diseases, including cardiovascular and neurological disorders (Beck and Offenbacher, 2005; Poole et al., 2013). Periodontitis is a chronic inflammatory disease caused by the abnormal growth and aggregation of different microorganisms (Kassebaum et al., 2014; Poole et al., 2015). In periodontitis, of the about 800 microorganisms identified so far, it appears that the vast majority of germs are Gram-positive (early colonizers), followed by Gram-negative bacteria (late colonizers). The latter are on the tooth surface, where they contribute to form the dental plaque (Belstrøm et al., 2014; Liccardo et al., 2019). These species include *Porphyromonas gingivalis*, *Aggregatibacter actinomycetemcomitans*, *Treponema denticola*, *Prevotella intermedia*, *Campylobacter rectus*, *Tannerella forsythia*, *Fusobacterium nucleatum*, *Selenomonas* spp., *Parvimonas micra*, and *Eubacterium timidum* (Socransky et al., 1998; Belstrøm et al., 2014; Liccardo et al., 2019). Interestingly, poor oral hygiene results in the increase of the anaerobic environment in the dental plaque, promoting the proliferation of these pathogens and the release of their toxic factors. Moreover, defects in host immunoregulation enable pathogen proliferation and increase local inflammation (Barth et al., 2013). Paradoxically, also neutrophils, i.e., the most efficient phagocytes and primary cellular defense recruited to the periodontal pocket, participate in the pathogenesis of periodontitis (Hajishengallis, 2015). Indeed, these immune cell types release several molecules (antimicrobial peptides, enzymes, and reactive oxygen species) that cannot discriminate between pathogens and host tissue. Moreover, certain agents, such as *P. gingivalis*, may subvert neutrophil function, inhibiting the phagocytosis, thus expanding the inflammatory response (Sochalska and Potempa, 2017). Because of this process, additional mediators and cytokines are produced, and more neutrophils, T cells, and monocytes are recruited to the periodontium, leading to chronic local and systemic inflammation (Cekici et al., 2014; Hajishengallis, 2014; Hajishengallis, 2015). Importantly, T cells promote the release of several cytokines and inflammatory mediators, including tumor necrosis factor alpha (TNF-α), interleukin (IL)-1, IL-4, IL-10, and transforming growth factor β (TGF-β) (Graves, 2008). In addition to these inflammatory mediators, in response to pathogen infection, the gingival epithelial cells and fibroblasts release other cytokines and mediators [i.e., IL-1, IL-8, TNF-α, and prostaglandin E2 (PGE2)] that, in turn, recruit more macrophages and neutrophils. Moreover, these cells promote the expression of matrix metalloproteinases (MMPs), tissue-derived enzymes that participate in the extracellular matrix remodeling. Altogether, these processes result in the stimulation of osteoclasts with subsequent alveolar bone reabsorption (Neely et al., 2005; Jin J. et al., 2014). Periodontitis leads to systemic inflammation due to the direct infiltration of bacteria and their virulence factors into the bloodstream (Poole et al., 2013). For this reason, periodontitis has been linked to the onset and progression of disorders systemically, such as cancer, diabetes, and cardiovascular and neurological diseases (Hajishengallis, 2015; Liccardo et al., 2019). Importantly, virulence factors expressed by periodontal pathogens are important pathogenic determinants in the initiation, progression, and severity of the disease, and they are responsible for the local and systemic inflammatory response observed in patients with periodontitis. For instance, *P. gingivalis*, long considered as one of the most important members of the periodontopathic microbiota, presents a specific LPS (LPS-Pg), which is recognized by immune cells *via* Toll-like receptors 2 and 4 (TLR2/4), and toxic proteases called gingipains (gps) and other surface components such as carbohydrates and fimbriae (Potempa et al., 1995, 1997; Holt et al., 1999; Imamura, 2003; Hasegawa et al., 2008; Yilmaz, 2008; Guo et al., 2010). gps are cysteine proteases that comprise lysine-gp (Kgp) and arginine-gp A (RgpA) and B (RgpB) are released and transported to the outer bacterial membrane surfaces (Guo et al., 2010). In synergy with other virulence factors (Guo et al., 2010; Dominy et al., 2019) these proteases are crucially involved in *P. gingivalis* survival and pathogenicity, allowing the colonization and invasion of gingival/periodontal tissues as well as other tissues, systemically. Importantly, the initial colonization of cells, including fibroblasts, epithelial cells, and other bacteria, is mostly mediated by the coordination between gps and the fimbrial and non-fimbrial components. Moreover, gps play a critical role in iron and nutrient acquisition (*P. gingivalis* agglutinates erythrocytes and lyses them to release hemoglobin), tissue destruction (Guo et al., 2010), and in the inactivation of host defenses escaping phagocytosis from immune cells (i.e., neutrophils) (Maekawa et al., 2014). Analogously, *A. actinomycetemcomitans* produces numerous factors that have been well characterized, including adherence proteins, LPS, and toxins like the cytolethal distending toxin (CDT) and leukotoxin (LtxA) (Kolodrubetz et al., 1989; Lally et al., 1989; Shenker et al., 2005). Of note is that these toxins are involved in immune evasion mechanisms (Kachlany, 2010). Finally, *T. forsythia* expresses several proteases that contribute to bacterial virulence in multiple manners. For example, proteases participate in degrading the host periodontal tissue, modifying host cell proteins, thus allowing bacterial colonization. Moreover, all the above-mentioned factors are able to activate host degradative enzymes that process components involved in innate and adaptive immunity, thus blocking the host immune response (Sharma, 2010).

## Relationship Between Periodontitis and Alzheimer’s Disease

Although the brain is considered an immune-isolated environment, several shreds of evidence have indicated that systemic inflammation contributes to neurodegeneration through the microglial activation and release of pro-inflammatory molecules, thus driving AD progression (Perry et al., 2007; Holmes, 2013). For instance, Capsoni and colleagues, in 2012, have demonstrated that pathogen-free conditions can delay the onset of neurodegeneration in a murine model of nerve growth factor (NGF) deprivation (Capsoni et al., 2012). Furthermore, LPS, the main component of the membrane of Gram-negative bacteria, can be found in large amounts in the brain of AD patients compared to healthy controls (Zhan et al., 2016). In this context, several studies have found that LPS co-localized with AβPs (Aβ40/42) in the amyloid plaques and around vessels of the brain of AD patients (Zhan et al., 2018). And peripheral injection of LPS in mice can activate microglia, inducing the release of pro-inflammatory cytokines, such as interleukins and TNF-α (Godbout et al., 2005). In line with these data, Sheng et al. (2003) demonstrated that mice infused with LPS presented an increased neuroinflammation associated with the enhanced expression and processing of APP and Aβ40/42 levels inside neurons. Lastly, Lee and colleagues showed that in rTg4510 mice expressing a mutated tau protein (TauP301L) that develop tauopathy between 3 and 5 months of age, LPS infusion increases microglial activation and tangle formations (Lee et al., 2010). Thus, these reports indicate that bacteria can induce local inflammatory damage, which, in chronic condition, is a trigger of neuroinflammation, constituting a significant contributor of neurodegeneration and AD. For this reason, periodontal pathogens have been investigated for their involvement in AD development and progression. For instance, Chen and colleagues have demonstrated, in a retrospective study, that periodontitis exposure is associated with an about 1.7-fold increase in the risk of developing AD (Chen et al., 2017). Analogously, a recent study analyzing the National Health and Nutrition Examination Survey (NHANES) database demonstrated that subjects with mild to severe periodontitis presented a decreased cognitive function compared with the healthy group (Sung et al., 2019). Mechanistically, this association has been demonstrated in a different number of studies. Kamer et al. (2009) have observed that elevated serum levels of TNF-α and serum antibodies to *P. gingivalis*, *A. actinomycetemcomitans*, and *T. forsythia* were present in AD patients compared to the controls. In line with these data, Sparks Stein and coworkers demonstrated that antibody levels to *F. nucleatum* and *P. intermedia*, at baseline, resulted significantly increased compared to the controls and correlated with a declined cognitive function in AD patients (Sparks et al., 2012). Furthermore, in a preclinical study from Ilievski and coworkers, it has been shown that in wild-type mice, *P. gingivalis* infection resulted in the neurodegeneration and formation of extracellular Aβ42 (Ilievski et al., 2018). Analogously, Díaz-Zúñiga et al. (2019) demonstrated that *in vitro* LPS (from *A. actinomycetemcomitans*) increased neuroinflammation *via* the activation of microglia and the subsequent increase in pro-inflammatory cytokines and chemokines coupled to the accumulation of Aβ42. Importantly, LPS from *P. gingivalis* (LPS-PG) binds to glial cells (Poole et al., 2013), and in the AD brain, it is co-localized with Aβ plaques (Zhan et al., 2016; Zhao et al., 2017). Of note is that a direct connection between oral dysbiosis and AD has been suggested by Poole et al. (2013), who reported the presence of periodontal pathogen components in AD subjects. Subsequently and in line with these data, Dominy et al. (2019) demonstrated that *P. gingivalis* and their virulence factors, gingipains, were exclusively detected in the brain of AD patients compared to the controls. Moreover, in this study, the authors demonstrated that in mice, this oral pathogen migrates from the mouth to the brain, increasing the production of Aβ42, exerting significant neurotoxic effects. Conversely, these processes were abolished following treatment with gingipain inhibitors. In this context, a phase II/III clinical trial has been designed and initiated in order to test the effects of the gingipain inhibitor COR388 in patients with a diagnosis of mild to moderate AD (NCT03823404).

Importantly, as discussed above, the ApoE genotype appears to be crucially involved in neuroinflammation, and as previously demonstrated, it can also contribute to enhancing *P. gingivalis* brain colonization. For example, in 2015, Poole and coworkers observed the presence of *P. gingivalis* DNA (Poole et al., 2015) in the brain of ApoE^null^ mice infected at gingival levels with this Gram-positive pathogen. Interestingly, as demonstrated by Singhrao et al., in these mice, gingival infection with *P. gingivalis* also resulted in the early appearance of age-related granules (Singhrao et al., 2015). These data, in line with the results obtained in another study by Hafezi-Moghadam et al., suggest that the lack of functional ApoE protein and the increased systemic inflammation, observed in periodontitis, induce an impairment of the BBB (Hafezi-Moghadam et al., 2007; Singhrao et al., 2017; Ranjan et al., 2018).

**FIGURE 2:**
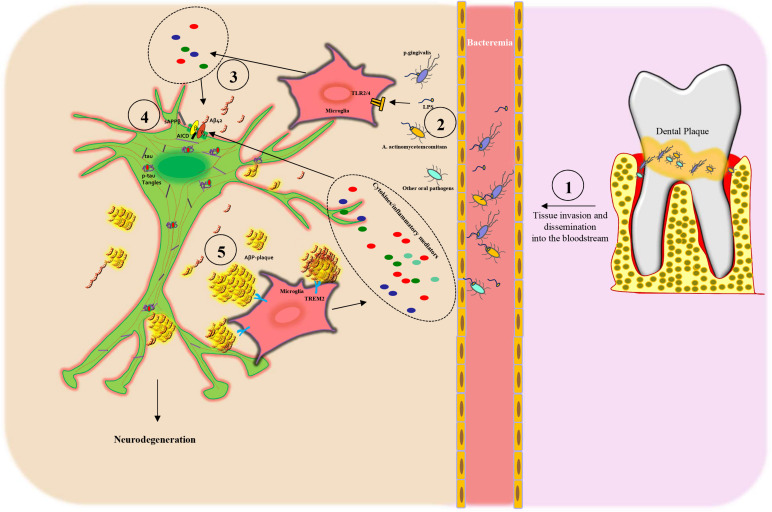
Scheme of the proposed mechanism linking periodontitis to Alzheimer’s disease: (1) Oral dysbiosis of the dental plaque leads to proliferation, tissue invasion, and then dissemination into the bloodstream of oral pathogens. (2) Next, the oral pathogens and their toxic molecules, such as lipopolysaccharide (LPS), bind to microglia *via* Toll-like receptors 2/4 (TLR2/4), inducing the release of cytokines (3) and inflammatory mediators that, in turn, lead to APP processing from neuronal cells. (3–4) Subsequently, the activation of β- and γ-secretase leads to an increased secretion of Aβ peptides (AβPs), in particular Aβ42 monomers and sAPPβ, outside of the cells and AICD intracellularly. (4) AβPs form oligomers, protofibrils, or fibrils and then amyloid plaques that are recognized by TREM2 receptors on the microglia plasma membrane, thus triggering an inflammatory response, which again stimulates AβP production. Dysfunctional neurons present also increased tau phosphorylation (p-tau) with the formation of neurofibrillary tangles (p-tau tangles). All these processes induce neuronal degeneration.

Importantly, a dysfunctional BBB allows periodontal pathogens to access the systemic circulation (bacteremia) and invade the brain (Hafezi-Moghadam et al., 2007; Singhrao et al., 2017; Ranjan et al., 2018) and represents an early feature of AD and cognitive decline (Van de Haar et al., 2016; Carter, 2017). In aggregate, these data strengthen the potential relationship between periodontitis and AD development and progression (Figure 2).

## Conclusion

In summary, periodontitis and AD often coexist. However, the current debate focuses on one main question: *what comes first*? Some studies have demonstrated that people with periodontitis present a major risk of developing AD (Chen et al., 2017); however, other reports suggest that those with AD or dementia suffer from inadequate oral health, stemming from cognitive decline, and are, therefore, more likely to develop periodontitis (Tada et al., 2006; Gonsalves et al., 2008; Maldonado et al., 2018). Thus, further studies are urgently needed to establish the *raison d’être* for the mutual association between periodontitis and AD. Along this line of reasoning, the trial (NCT03823404) discussed above shall give us the proof-of-concept of the beneficial role of oral pathogen blockade in human AD. Yet, while waiting for the publication of the trial outcome, we can ascertain, with no additional hesitation, that a more careful dental treatment effectively improves the quality of life/cognitive impairment of patients with mild AD (Rolim et al., 2014). Likewise, in decreasing the incidence of dementia in patients treated for dementia or periodontitis (Lee et al., 2017; Yoo et al., 2019). Therefore, oral hygiene care strategies should be included in the routine health care of patients with dementia and cognitive impairment and become a dominant part of adult oral health programs to avoid any extra-neuronal source of inflammation as well as to prevent the onset of neurodegeneration. Thus, these findings highlight the necessity to prevent the progression of periodontitis and encourage healthcare service at the national level.

## Author Contributions

DL wrote, edited, and revised the manuscript. FM, FC, MG, GF, LB, JA, AA, and IM contributed to the writing and editing of the manuscript. AV, CR, NF, and GR revised the manuscript. AC supervised the project, revised the manuscript and generated the figures.

## Conflict of Interest

The authors declare that the research was conducted in the absence of any commercial or financial relationships that could be construed as a potential conflict of interest.

## References

[B1] Agosta F. Vossel K. A. Miller B. L. Migliaccio R. Bonasera S. J. Filippi M. (2009). Apolipoprotein E ε4 is associated with disease-specific effects on brain atrophy in Alzheimer’s disease and frontotemporal dementia. *Proc. Natl. Acad. Sci. U.S.A.* 106 2018–2022. 10.1073/pnas.0812697106 19164761 PMC2644156

[B2] Alexandraki K. I. Apostolopoulos N. V. Adamopoulos C. Stamouli E. Dalagiorgou G. Papaioannou T. G. (2019). Differential expression of apoptotic and low-grade inflammatory markers in alzheimer disease compared to diabetes mellitus type 1 and 2. *J. Appl. Lab. Med.* 3 1003–1013. 10.1373/jalm.2018.027623 31639691

[B3] Ali M. M. Ghouri R. G. Ans A. H. Akbar A. Toheed A. (2019). Recommendations for Anti-inflammatory Treatments in Alzheimer’s disease: a comprehensive review of the literature. *Cureus* 11:e4620. 10.7759/cureus.4620 31312547 PMC6615583

[B4] Almansoub H. A. M. M. Tang H. Wu Y. Wang D. Q. Mahaman Y. A. R. Wei N. (2019). Tau abnormalities and the potential therapy in Alzheimer’s disease. *J. Alzheimers. Dis.* 67 13–33. 10.3233/JAD-180868 30507581

[B5] Alzheimer A. Stelzmann R. A. Schnitzlein H. N. Murtagh F. R. (1995). An english translation of Alzheimer’s 1907 paper, “Uber eine eigenartige Erkankung der Hirnrinde”. *Clin. Anat.* 8 429–431. 10.1002/ca.980080612 8713166

[B6] Alzheimer’s Association. (2016). Alzheimer’s disease facts and figures. *Alzheimers Dement.* 12 459–509. 10.1016/j.jalz.2016.03.001 27570871

[B7] Andersson C. Blennow K. Almkvist O. Andreasen N. Engfeldt P. Johansson S. E. (2008). Increasing CSF phospho-tau levels during cognitive decline and progression to dementia. *Neurobiol. Aging* 29 1466–1473. 10.1016/j.neurobiolaging.2007.03.027 17512092

[B8] Andrew R. J. Kellett K. A. Thinakaran G. Hooper N. M. (2016). A greek tragedy: the growing complexity of alzheimer amyloid precursor protein proteolysis. *J. Biol. Chem.* 291 19235–19244. 10.1074/jbc.R116.746032 27474742 PMC5016663

[B9] Atagi Y. Liu C. C. Painter M. M. Chen X. F. Verbeeck C. Zheng H. (2015). Apolipoprotein E Is a ligand for triggering receptor expressed on myeloid cells 2 (TREM2). *J. Biol. Chem.* 290 26043–26050. 10.1074/jbc.M115.679043 26374899 PMC4646257

[B10] Barth K. Remick D. G. Genco C. A. (2013). Disruption of immune regulation by microbial pathogens and resulting chronic inflammation. *J. Cell. Physiol.* 228 1413–1422. 10.1002/jcp.24299 23255141 PMC3995356

[B11] Beck J. D. Offenbacher S. (2005). Systemic effects of periodontitis: epidemiology of periodontal disease and cardiovascular disease. *J. Periodontol.* 76 2089–2100. 10.1902/jop.2005.76.11-S.2089 16277581

[B12] Bekris L. M. Yu C. E. Bird T. D. Tsuang D. W. (2010). Genetics of Alzheimer disease. *J. Geriatr. Psychiatry Neurol.* 23 213–227. 10.1177/0891988710383571 21045163 PMC3044597

[B13] Belstrøm D. Fiehn N. E. Nielsen C. H. Kirkby N. Twetman S. Klepac-Ceraj V. (2014). Differences in bacterial saliva profile between periodontitis patients and a control cohort. *J. Clin. Periodontol.* 41 104–112. 10.1111/jcpe.12190 24303924

[B14] Belyaev N. D. Kellett K. A. Beckett C. Makova N. Z. Revett T. J. Nalivaeva N. N. (2010). The transcriptionally active amyloid precursor protein (APP) intracellular domain is preferentially produced from the 695 isoform of APP in a β-secretase-dependent pathway. *J. Biol. Chem.* 285 41443–41454. 10.1074/jbc.M110.141390 20961856 PMC3009870

[B15] Ben Halima S. Mishra S. Raja K. M. P. Willem M. Baici A. Simons K. (2016). Specific inhibition of β-secretase processing of the Alzheimer disease amyloid precursor protein. *Cell Rep.* 14 2127–2141. 10.1016/j.celrep.2016.01.076 26923602

[B16] Boche D. Perry V. H. Nicoll J. A. (2013). Review: activation patterns of microglia and their identification in the human brain. *Neuropathol. Appl. Neurobiol.* 39 3–18. 10.1111/nan.12011 23252647

[B17] Bornemann K. D. Wiederhold K. H. Pauli C. Ermini F. Stalder M. Schnell L. (2001). Abeta-induced inflammatory processes in microglia cells of APP23 transgenic mice. *Am. J. Pathol.* 158 63–73. 10.1016/s0002-9440(10)63945-4 11141480 PMC1850262

[B18] Braak H. Braak E. (1995). Staging of Alzheimer’s disease-related neurofibrillary changes. *Neurobiol. Aging* 16 271–278; discussion 278–284 10.1016/0197-4580(95)00021-6 7566337

[B19] Brookmeyer R. Corrada M. M. Curriero F. C. Kawas C. (2002). Survival following a diagnosis of Alzheimer disease. *Arch. Neurol.* 59 1764–1767. 12433264 10.1001/archneur.59.11.1764

[B20] Brookmeyer R. Johnson E. Ziegler-Graham K. Arrighi H. M. (2007). Forecasting the global burden of Alzheimer’s disease. *Alzheimers Dement.* 3 186–191. 10.1016/j.jalz.2007.04.381 19595937

[B21] Brouwers N. Van Cauwenberghe C. Engelborghs S. Lambert J. C. Bettens K. Le Bastard N. (2012). Alzheimer risk associated with a copy number variation in the complement receptor 1 increasing C3b/C4b binding sites. *Mol. Psychiatry* 17 223–233. 10.1038/mp.2011.24 21403675 PMC3265835

[B22] Buerger K. Ewers M. Pirttilä T. Zinkowski R. Alafuzoff I. Teipel S. J. (2006). CSF phosphorylated tau protein correlates with neocortical neurofibrillary pathology in Alzheimer’s disease. *Brain* 129(Pt 11), 3035–3041. 10.1093/brain/awl269 17012293

[B23] Bui F. Q. Almeida-da-Silva C. L. C. Huynh B. Trinh A. Liu J. Woodward J. (2019). Association between periodontal pathogens and systemic disease. *Biomed. J.* 42 27–35. 10.1016/j.bj.2018.12.001 30987702 PMC6468093

[B24] Cao W. Zheng H. (2018). Peripheral immune system in aging and Alzheimer’s disease. *Mol. Neurodegener.* 13:51. 10.1186/s13024-018-0284-2 PMC6169078 30285785

[B25] Capsoni S. Carucci N. M. Cattaneo A. (2012). Pathogen free conditions slow the onset of neurodegeneration in a mouse model of nerve growth factor deprivation. *J. Alzheimers Dis.* 31 1–6. 10.3233/jad-2012-120427 22504318

[B26] Carbone I. Lazzarotto T. Ianni M. Porcellini E. Forti P. Masliah E. (2014). Herpes virus in Alzheimer’s disease: relation to progression of the disease. *Neurobiol. Aging* 35 122–129. 10.1016/j.neurobiolaging.2013.06.024 23916950

[B27] Carter C. J. (2017). Genetic, transcriptome, proteomic and epidemiological evidence for blood-brain barrier disruption and polymicrobial brain invasion as determinant factors in Alzheimer’s disease. *J. Alzheimers Dis. Rep.* 1 125–157. 10.1101/080333 30480234 PMC6159731

[B28] Cekici A. Kantarci A. Hasturk H. Van Dyke T. E. (2014). Inflammatory and immune pathways in the pathogenesis of periodontal disease. *Periodontology* 64 57–80. 10.1111/prd.12002 24320956 PMC4500791

[B29] Chen C. K. Wu Y. T. Chang Y. C. (2017). Association between chronic periodontitis and the risk of Alzheimer’s disease: a retrospective, population-based, matched-cohort study. *Alzheimers Res. Ther.* 9:56. 10.1186/s13195-017-0282-6 28784164 PMC5547465

[B30] Chen V. C. Wu S. I. Huang K. Y. Yang Y. H. Kuo T. Y. Liang H. Y. (2018). Herpes zoster and dementia: a nationwide population-based cohort study. *J. Clin. Psychiatry* 79:16m11312. 10.4088/JCP.16m11312 29244265

[B31] Cheng-Hathaway P. J. Reed-Geaghan E. G. Jay T. R. Casali B. T. Bemiller S. M. Puntambekar S. S. (2018). The Trem2 R47H variant confers loss-of-function-like phenotypes in Alzheimer’s disease. *Mol. Neurodegener.* 13:29. 10.1186/s13024-018-0262-8 PMC5984804 29859094

[B32] Cleveland D. W. Hwo S. Y. Kirschner M. W. (1977). Purification of tau, a microtubule-associated protein that induces assembly of microtubules from purified tubulin. *J. Mol. Biol.* 116 207–225. 10.1016/0022-2836(77)90213-3 599557

[B33] Colombo E. Farina C. (2016). Astrocytes: key regulators of neuroinflammation. *Trends Immunol.* 37 608–620. 10.1016/j.it.2016.06.006 27443914

[B34] Congdon E. E. Sigurdsson E. M. (2018). Tau-targeting therapies for Alzheimer disease. *Nat. Rev. Neurol.* 14 399–415. 10.1038/s41582-018-0013-z 29895964 PMC6463489

[B35] Corder E. H. Saunders A. M. Strittmatter W. J. Schmechel D. E. Gaskell P. C. Small G. W. (1993). Gene dose of apolipoprotein E type 4 allele and the risk of Alzheimer’s disease in late onset families. *Science* 261 921–923. 10.1126/science.8346443 8346443

[B36] Davis M. O. Connell T. Johnson S. Cline S. Merikle E. Martenyi F. (2018). Estimating Alzheimer’s disease progression rates from normal cognition through mild cognitive impairment and stages of dementia. *Curr. Alzheimer Res.* 15 777–788. 10.2174/1567205015666180119092427 29357799 PMC6156780

[B37] de Leon M. J. DeSanti S. Zinkowski R. Mehta P. D. Pratico D. Segal S. (2004). MRI and CSF studies in the early diagnosis of Alzheimer’s disease. *J. Int. Med.* 256 205–223. 10.1111/j.1365-2796.2004.01381.x 15324364

[B38] De Strooper B. Iwatsubo T. Wolfe M. S. (2012). Presenilins and γ-secretase: structure, function, and role in Alzheimer disease. *Cold Spring Harb. Perspect. Med.* 2:a006304. 10.1101/cshperspect.a006304 22315713 PMC3253024

[B39] De Strooper B. Saftig P. Craessaerts K. Vanderstichele H. Guhde G. Annaert W. (1998). Deficiency of presenilin-1 inhibits the normal cleavage of amyloid precursor protein. *Nature* 391 387–390. 10.1038/34910 9450754

[B40] Díaz-Zúñiga J. Muñoz Y. Melgar-Rodríguez S. More J. Bruna B. Lobos P. (2019). Serotype b of Aggregatibacter actinomycetemcomitans triggers pro-inflammatory responses and amyloid beta secretion in hippocampal cells: a novel link between periodontitis and Alzheimer’s disease? *J. Oral Microbiol.* 11:1586423. 10.1080/20002297.2019.1586423 31044031 PMC6484476

[B41] Dominy S. S. Lynch C. Ermini F. Benedyk M. Marczyk A. Konradi A. (2019). Porphyromonas gingivalis in Alzheimer’s disease brains: evidence for disease causation and treatment with small-molecule inhibitors. *Sci. Adv.* 5:eaau3333. 10.1126/sciadv.aau3333 30746447 PMC6357742

[B42] Donev R. Kolev M. Millet B. Thome J. (2009). Neuronal death in Alzheimer’s disease and therapeutic opportunities. *J. Cell. Mol. Med.* 13 4329–4348. 10.1111/j.1582-4934.2009.00889.x 19725918 PMC4515050

[B43] Dorszewska J. Prendecki M. Oczkowska A. Dezor M. Kozubski W. (2016). Molecular basis of familial and sporadic Alzheimer’s disease. *Curr. Alzheimer Res.* 13 952–963. 10.2174/1567205013666160314150501 26971934

[B44] Drewes G. Trinczek B. Illenberger S. Biernat J. Schmitt-Ulms G. (1995). Microtubule-associated protein/microtubule affinity-regulating kinase (p110mark). A novel protein kinase that regulates tau-microtubule interactions and dynamic instability by phosphorylation at the Alzheimer-specific site serine 262. *J. Biol. Chem.* 270 7679–7688. 10.1074/jbc.270.13.7679 7706316

[B45] Du L. Zhang Y. Chen Y. Zhu J. Yang Y. Zhang H. L. (2017). Role of microglia in neurological disorders and their potentials as a therapeutic target. *Mol. Neurobiol.* 54 7567–7584. 10.1007/s12035-016-0245-0 27830532

[B46] Ellis R. J. Olichney J. M. Thal L. J. Mirra S. S. Morris J. C. Beekly D. (1996). Cerebral amyloid angiopathy in the brains of patients with Alzheimer’s disease: the CERAD experience. Part XV. *Neurology* 46 1592–1596. 10.1212/wnl.46.6.1592 8649554

[B47] Ewers M. Franzmeier N. Suárez-Calvet M. Morenas-Rodriguez E. Caballero M. A. A. Kleinberger G. (2019). Increased soluble TREM2 in cerebrospinal fluid is associated with reduced cognitive and clinical decline in Alzheimer’s disease. *Sci. Transl. Med.* 11:eaav6221. 10.1126/scitranslmed.aav6221 31462511 PMC7050285

[B48] Ezzat K. Pernemalm M. Pålsson S. Roberts T. C. Järver P. Dondalska A. (2019). The viral protein corona directs viral pathogenesis and amyloid aggregation. *Nat. Commun.* 10:2331. 10.1038/s41467-019-10192-2 31133680 PMC6536551

[B49] Femminella G. D. Dani M. Wood M. Fan Z. Calsolaro V. Atkinson R. (2019). Microglial activation in early Alzheimer trajectory is associated with higher gray matter volume. *Neurology* 92 e1331–e1343. 10.1212/WNL.0000000000007133 30796139 PMC6511099

[B50] Femminella G. D. Thayanandan T. Calsolaro V. Komici K. Rengo G. Corbi G. (2018). Imaging and molecular mechanisms of Alzheimer’s disease: a review. *Int. J. Mol. Sci.* 19:E3702. 10.3390/ijms19123702 30469491 PMC6321449

[B51] Fischer B. Popa-Wagner A. (1996). Alzheimer disease: involvement of the complement system in cell death. Gene expression of C1q and C3 in the frontal cortex of patients with Alzheimer disease and control probands. *Fortschr. Med.* 114 161–163. 8964559

[B52] Fischer B. Schmoll H. Riederer P. Bauer J. Platt D. Popa-Wagner A. (1995). Complement C1q and C3 mRNA expression in the frontal cortex of Alzheimer’s patients. *J. Mol. Med.* 73 465–471. 8528750 10.1007/BF00202265

[B53] Garwood C. J. Pooler A. M. Atherton J. Hanger D. P. Noble W. (2011). Astrocytes are important mediators of Aβ-induced neurotoxicity and tau phosphorylation in primary culture. *Cell Death Dis.* 2:e167. 10.1038/cddis.2011.50 21633390 PMC3168992

[B54] Gendron T. F. Petrucelli L. (2009). The role of tau in neurodegeneration. *Mol. Neurodegener.* 4:13. 10.1186/1750-1326-4-13 19284597 PMC2663562

[B55] Ghani M. Sato C. Kakhki E. G. Gibbs J. R. Traynor B. St George-Hyslop P. (2016). Mutation analysis of the MS4A and TREM gene clusters in a case-control Alzheimer’s disease data set. *Neurobiol. Aging* 42 217.e7–217.e13. 10.1016/j.neurobiolaging.2016.03.009 27084067 PMC8985522

[B56] Glenner G. G. Wong C. W. (1984). Alzheimer’s disease: initial report of the purification and characterization of a novel cerebrovascular amyloid protein. *Biochem. Biophys. Res. Commun.* 120 885–890. 10.1016/s0006-291x(84)80190-4 6375662

[B57] Godbout J. P. Chen J. Abraham J. Richwine A. F. Berg B. M. Kelley K. W. (2005). Exaggerated neuroinflammation and sickness behavior in aged mice following activation of the peripheral innate immune system. *FASEB J.* 19 1329–1331. 10.1096/fj.05-3776fje 15919760

[B58] Goldeck D. Witkowski J. M. Fülop T. Pawelec G. (2016). Peripheral immune signatures in Alzheimer disease. *Curr. Alzheimer Res.* 13 739–749. 10.2174/1567205013666160222112444 26899580

[B59] Gonsalves W. C. Wrightson A. S. Henry R. G. (2008). Common oral conditions in older persons. *Am. Fam. Phys.* 78 845–852. 18841733

[B60] Gratuze M. Leyns C. E. G. Holtzman D. M. (2018). New insights into the role of TREM2 in Alzheimer’s disease. *Mol. Neurodegener.* 13:66. 10.1186/s13024-018-0298-9 PMC6302500 30572908

[B61] Graves D. (2008). Cytokines that promote periodontal tissue destruction. *J. Periodontol.* 79 8(Suppl.), 1585–1591. 10.1902/jop.2008.080183 18673014

[B62] Griciuc A. Patel S. Federico A. N. Choi S. H. Innes B. J. Oram M. K. (2019). TREM2 Acts downstream of CD33 in modulating microglial pathology in Alzheimer’s disease. *Neuron* 103 820–835.e7. 10.1016/j.neuron.2019.06.010 31301936 PMC6728215

[B63] Griciuc A. Serrano-Pozo A. Parrado A. R. Lesinski A. N. Asselin C. N. Mullin K. (2013). Alzheimer’s disease risk gene CD33 inhibits microglial uptake of amyloid beta. *Neuron* 78 631–643. 10.1016/j.neuron.2013.04.014 23623698 PMC3706457

[B64] Grimm M. O. Mett J. Stahlmann C. P. Grösgen S. Haupenthal V. J. Blümel T. (2015). APP intracellular domain derived from amyloidogenic β- and γ-secretase cleavage regulates neprilysin expression. *Front. Aging Neurosci.* 7:77. 10.3389/fnagi.2015.00077 26074811 PMC4443740

[B65] Gu Y. Misonou H. Sato T. Dohmae N. Takio K. Ihara Y. (2001). Distinct intramembrane cleavage of the β-amyloid precursor protein family resembling γ-secretase-like cleavage of Notch. *J. Biol. Chem.* 276 35235–35238. 10.1074/jbc.c100357200 11483588

[B66] Guerreiro R. Wojtas A. Bras J. Carrasquillo M. Rogaeva E. Majounie E. (2013). TREM2 variants in Alzheimer’s disease. *N. Engl. J. Med.* 368 117–127. 10.1056/NEJMoa1211851 23150934 PMC3631573

[B67] Gulisano W. Maugeri D. Baltrons M. A. Fà M. Amato A. Palmeri A. (2018). Role of Amyloid-β and tau proteins in Alzheimer’s disease: Confuting the amyloid cascade. *J. Alzheimers Dis.* 64 S611–S631. 10.3233/JAD-179935 29865055 PMC8371153

[B68] Guo Y. Nguyen K. A. Potempa J. (2010). Dichotomy of gingipains action as virulence factors: from cleaving substrates with the precision of a surgeon’s knife to a meat chopper-like brutal degradation of proteins. *Periodontology* 54 15–44. 10.1111/j.1600-0757.2010.00377.x 20712631 PMC2924770

[B69] Haass C. Selkoe D. J. (2007). Soluble protein oligomers in neurodegeneration: Lessons from the Alzheimer’s amyloid beta-peptide. *Nat. Rev. Mol. Cell Biol.* 8 101–112. 10.1038/nrm2101 17245412

[B70] Hafezi-Moghadam A. Thomas K. L. Wagner D. D. (2007). ApoE deficiency leads to a progressive age-dependent blood-brain barrier leakage. *Am. J. Physiol. Cell Physiol.* 292 C1256–C1262. 16870825 10.1152/ajpcell.00563.2005

[B71] Hajishengallis G. (2014). Immunomicrobial pathogenesis of periodontitis: keystones, pathobionts, and host response. *Trends Immunol.* 35 3–11. 10.1016/j.it.2013.09.001 24269668 PMC3947349

[B72] Hajishengallis G. (2015). Periodontitis: from microbial immune subversion to systemic inflammation. *Nat. Rev. Immunol.* 15 30–44. 10.1038/nri3785 25534621 PMC4276050

[B73] Hansen D. V. Hanson J. E. Sheng M. (2018). Microglia in Alzheimer’s disease. *J. Cell Biol.* 217 459–472. 10.1083/jcb.201709069 29196460 PMC5800817

[B74] Hasegawa Y. Tribble G. D. Baker H. V. Mans J. J. Handfield M. Lamont R. J. (2008). Role of *Porphyromonas gingivalis* SerB in gingival epithelial cell cytoskeletal remodeling and cytokine production. *Infect. Immun.* 76 2420–2427. 10.1128/iai.00156-08 18391005 PMC2423092

[B75] Hayden E. Y. Teplow D. B. (2013). Amyloid β-protein oligomers and Alzheimer’s disease. *Alzheimers Res. Ther.* 5:60. 10.1186/alzrt226 24289820 PMC3978746

[B76] Head D. Bugg J. M. Goate A. M. Fagan A. M. Mintun M. A. Benzinger T. (2012). Exercise engagement as a moderator of the effects of APOE genotype on amyloid deposition. *Arch. Neurol.* 69 636–643. 22232206 10.1001/archneurol.2011.845 PMC3583203

[B77] Heppner F. L. Ransohoff R. M. Becher B. (2015). Immune attack: the role of inflammation in Alzheimer disease. *Nat. Rev. Neurosci.* 16 358–372. 10.1038/nrn3880 25991443

[B78] Holmes C. (2013). Review: systemic inflammation and Alzheimer’s disease. *Neuropathol. Appl. Neurobiol.* 39 51–68. 10.1111/j.1365-2990.2012.01307.x 23046210

[B79] Holt S. C. Kesavalu L. Walker S. Genco C. A. (1999). Virulence factors of *Porphyromonas gingivalis*. *Periodontology* 20 168–238. 10.1111/j.1600-0757.1999.tb00162.x 10522227

[B80] Huang Y. Weisgraber K. H. Mucke L. Mahley R. W. (2004). Apolipoprotein E: diversity of cellular origins, structural and biophysical properties, and effects in Alzheimer’s disease. *J. Mol. Neurosci.* 23 189–204. 10.1385/jmn:23:3:189 15181247

[B81] Ilievski V. Zuchowska P. K. Green S. J. Toth P. T. Ragozzino M. E. Le K. (2018). Chronic oral application of a periodontal pathogen results in brain inflammation, neurodegeneration and amyloid beta production in wild type mice. *PLoS ONE* 13:e0204941. 10.1371/journal.pone.0204941 30281647 PMC6169940

[B82] Imamura T. (2003). The role of gingipains in the pathogenesis of periodontal disease. *J. Periodontol.* 74 111–118. 10.1902/jop.2003.74.1.111 12593605

[B83] Jamieson G. A. Maitland N. J. Wilcock G. K. Craske J. Itzhaki R. F. (1991). Latent herpes simplex virus type 1 in normal and Alzheimer’s disease brains. *J. Med. Virol.* 33 224–227. 10.1002/jmv.1890330403 1649907

[B84] Jamieson G. A. Maitland N. J. Wilcock G. K. Yates C. M. Itzhaki R. F. (1992). Herpes simplex virus type 1 DNA is present in specific regions of brain from aged people with and without senile dementia of the Alzheimer type. *J. Pathol.* 167 365–368. 10.1002/path.1711670403 1328575

[B85] Jendresen C. Årskog V. Daws M. R. Nilsson L. N. (2017). The Alzheimer’s disease risk factors apolipoprotein E and TREM2 are linked in a receptor signaling pathway. *J. Neuroinflamm.* 14:59. 10.1186/s12974-017-0835-4 28320424 PMC5360024

[B86] Jevtic S. Sengar A. S. Salter M. W. McLaurin J. (2017). The role of the immune system in Alzheimer disease: etiology and treatment. *Ageing Res. Rev.* 40 84–94. 10.1016/j.arr.2017.08.005 28941639

[B87] Jin S. C. Benitez B. A. Karch C. M. Cooper B. Skorupa T. Carrell D. (2014). Coding variants in TREM2 increase risk for Alzheimer’s disease. *Hum. Mol. Genet.* 23 5838–5846. 10.1093/hmg/ddu277 24899047 PMC4189899

[B88] Jin J. Zhang X. Lu Z. Li Y. Lopes-Virella M. F. Yu H. (2014). Simvastatin inhibits lipopolysaccharide-induced osteoclastogenesis and reduces alveolar bone loss in experimental periodontal disease. *J. Periodontal Res.* 49 518–526. 10.1111/jre.12132 24117880 PMC3979522

[B89] Jonsson T. Stefansson H. Steinberg S. Jonsdottir I. Jonsson P. V. Snaedal J. (2013). Variant of TREM2 associated with the risk of Alzheimer’s disease. *N. Engl. J. Med.* 368 107–116. 10.1056/NEJMoa1211103 23150908 PMC3677583

[B90] Kachlany S. C. (2010). Aggregatibacter actinomycetemcomitans leukotoxin: from threat to therapy. *J. Dent. Res.* 89 561–570. 10.1177/0022034510363682 20200418 PMC3144085

[B91] Kakuda N. Funamoto S. Yagishita S. Takami M. Osawa S. Dohmae N. (2006). Equimolar production of amyloid β-protein and amyloid precursor protein intracellular domain from β-carboxyl-terminal fragment by γ-secretase. *J. Biol. Chem.* 281 14776–11478. 16595682 10.1074/jbc.M513453200

[B92] Kamer A. R. Craig R. G. Dasanayake A. P. Brys M. Glodzik-Sobanska L. de Leon M. J. (2008). Inflammation and Alzheimer’s disease: possible role of periodontal diseases. *Alzheimers Dement.* 4 242–250. 10.1016/j.jalz.2007.08.004 18631974

[B93] Kamer A. R. Craig R. G. Pirraglia E. Dasanayake A. P. Norman R. G. Boylan R. J. (2009). TNF-alpha and antibodies to periodontal bacteria discriminate between Alzheimer’s disease patients and normal subjects. *J. Neuroimmunol.* 216 92–97. 10.1016/j.jneuroim.2009.08.013 19767111 PMC2783848

[B94] Kassebaum N. J. Bernabé E. Dahiya M. Bhandari B. Murray C. J. Marcenes W. (2014). Global burden of severe periodontitis in 1990-2010: a systematic review and meta-regression. *J. Dent. Res.* 93 1045–1053. 10.1177/0022034514552491 25261053 PMC4293771

[B95] Kettenmann H. Hanisch U. K. Noda M. Verkhratsky A. (2011). Physiology of microglia. *Physiol. Rev.* 91 461–553. 10.1152/physrev.00011.2010 21527731

[B96] Kim J. Amar S. (2006). Periodontal disease and systemic conditions: a bidirectional relationship. *Odontology* 94 10–21. 10.1007/s10266-006-0060-6 16998613 PMC2443711

[B97] Kinney J. W. Bemiller S. M. Murtishaw A. S. Leisgang A. M. Salazar A. M. Lamb B. T. (2018). Inflammation as a central mechanism in Alzheimer’s disease. *Alzheimers Dement (N Y)* 4 575–590. 10.1016/j.trci.2018.06.014 30406177 PMC6214864

[B98] Kleinberger G. Yamanishi Y. Suárez-Calvet M. Czirr E. Lohmann E. Cuyvers E. (2014). TREM2 mutations implicated in neurodegeneration impair cell surface transport and phagocytosis. *Sci. Transl. Med.* 6:243ra86. 10.1126/scitranslmed.3009093 24990881

[B99] Kok E. Haikonen S. Luoto T. Huhtala H. Goebeler S. Haapasalo H. (2009). Apolipoprotein E-dependent accumulation of Alzheimer disease-related lesions begins in middle age. *Ann. Neurol.* 65 650–657. 10.1002/ana.21696 19557866

[B100] Kolodrubetz D. Dailey T. Ebersole J. Kraig E. (1989). Cloning and expression of the leukotoxin gene from *Actinobacillus actinomycetemcomitans*. *Infect. Immun.* 57 1465–1469. 10.1128/iai.57.5.1465-1469.1989 2707855 PMC313300

[B101] Kucukkilic E. Brookes K. Barber I. Guetta-Baranes T. Aruk Consortium, Morgan K. (2018). Complement receptor 1 gene (CR1) intragenic duplication and risk of Alzheimer’s disease. *Hum. Genet.* 137 305–314. 10.1007/s00439-018-1883-2 29675612 PMC5937907

[B102] LaDu M. J. Shah J. A. Reardon C. A. Getz G. S. Bu G. Hu J. (2001). Apolipoprotein E and apolipoprotein E receptors modulate A beta-induced glial neuroinflammatory responses. *Neurochem. Int.* 39 427–434. 10.1016/s0197-0186(01)00050-x 11578778

[B103] Lagarde J. Sarazin M. Bottlaender M. (2018). In vivo PET imaging of neuroinflammation in Alzheimer’s disease. *J. Neural Transm.* 125 847–867. 10.1007/s00702-017-1731-x 28516240

[B104] Lally E. T. Golub E. E. Kieba I. R. Taichman N. S. Rosenbloom J. Rosenbloom J. C. (1989). Analysis of the *Actinobacillus actinomycetemcomitans* leukotoxin gene. Delineation of unique features and comparison to homologous toxins. *J. Biol. Chem.* 264 15451–15456. 2670940

[B105] Lambert J. C. Heath S. Even G. Campion D. Sleegers K. Hiltunen M. (2009). Genome-wide association study identifies variants at CLU and CR1 associated with Alzheimer’s disease. *Nat. Genet.* 41 1094–1099. 10.1038/ng.439 19734903

[B106] LaPointe N. E. Morfini G. Pigino G. Gaisina I. N. Kozikowski A. P. Binder L. I. (2009). The amino terminus of tau inhibits kinesin-dependent axonal transport: implications for filament toxicity. *J. Neurosci. Res.* 87 440–451. 10.1002/jnr.21850 18798283 PMC2739042

[B107] Lee D. C. Rizer J. Selenica M. L. Reid P. Kraft C. Johnson A. (2010). LPS- induced inflammation exacerbates phospho-tau pathology in rTg4510 mice. *J. Neuroinflamm.* 7:56. 10.1186/1742-2094-7-56 20846376 PMC2949628

[B108] Lee Y. L. Hu H. Y. Huang L. Y. Chou P. Chu D. (2017). Periodontal disease associated with higher risk of dementia: population-based cohort study in Taiwan. *J. Am. Geriatr. Soc.* 65 1975–1980. 10.1111/jgs.14944 28598507

[B109] Liccardo D. Cannavo A. Spagnuolo G. Ferrara N. Cittadini A. Rengo C. (2019). Periodontal disease: a risk factor for diabetes and cardiovascular disease. *Int. J. Mol. Sci.* 20:E1414. 10.3390/ijms20061414 30897827 PMC6470716

[B110] Liu C. C. Liu C. C. Kanekiyo T. Xu H. Bu G. (2013). Apolipoprotein E and Alzheimer disease: risk, mechanisms and therapy. *Nat. Rev. Neurol.* 9 106–118. 10.1038/nrneurol.2012.263 23296339 PMC3726719

[B111] Liu F. Shi J. Tanimukai H. Gu J. Gu J. Grundke-Iqbal I. (2009). Reduced O-GlcNAcylation links lower brain glucose metabolism and tau pathology in Alzheimer’s disease. *Brain* 132(Pt 7), 1820–1832. 10.1093/brain/awp099 19451179 PMC2702834

[B112] Long J. M. Holtzman D. M. (2019). Alzheimer disease: an update on pathobiology and treatment strategies. *Cell* 179 312–339. 10.1016/j.cell.2019.09.001 31564456 PMC6778042

[B113] Lynch J. R. Tang W. Wang H. Vitek M. P. Bennett E. R. Sullivan P. M. (2003). APOE genotype and an ApoE-mimetic peptide modify the systemic and central nervous system inflammatory response. *J. Biol. Chem.* 278 48529–48533. 10.1074/jbc.m306923200 14507923

[B114] Maekawa T. Krauss J. L. Abe T. Jotwani R. Triantafilou M. Triantafilou K. (2014). Porphyromonas gingivalis manipulates complement and TLR signaling to uncouple bacterial clearance from inflammation and promote dysbiosis. *Cell Host Microbe* 15 768–778. 10.1016/j.chom.2014.05.012 24922578 PMC4071223

[B115] Mahley R. W. Rall S. C. Jr. (2000). Apolipoprotein E: far more than a lipid transport protein. *Annu. Rev. Genomics Hum. Genet.* 1 507–537. 10.1146/annurev.genom.1.1.507 11701639

[B116] Maldonado A. Laugisch O. Bürgin W. Sculean A. Eick S. (2018). Clinical periodontal variables in patients with and without dementia-a systematic review and meta-analysis. *Clin. Oral Investig.* 22 2463–2474. 10.1007/s00784-018-2523-x 29934798

[B117] Mandrekar S. Landreth G. E. (2010). Microglia and inflammation in Alzheimer’s Disease. *CNS Neurol. Disord. Drug Targets* 9 156–167. 20205644 10.2174/187152710791012071 PMC3653290

[B118] Marchini L. Ettinger R. Caprio T. Jucan A. (2019). Oral health care for patients with Alzheimer’s disease: an update. *Spec Care Dentist.* 39 262–273. 10.1111/scd.12375 30964560

[B119] Martin L. Latypova X. Wilson C. M. Magnaudeix A. Perrin M. L. Yardin C. (2013). Tau protein kinases: involvement in Alzheimer’s disease. *Ageing Res. Rev.* 12 289–309. 10.1016/j.arr.2012.06.003 22742992

[B120] Mayer F. Di Pucchio A. Lacorte E. Bacigalupo I. Marzolini F. Ferrante G. (2018). An estimate of attributable cases of alzheimer disease and vascular dementia due to modifiable risk factors: the impact of primary prevention in europe and in italy. *Dement Geriatr. Cogn. Dis. Extra* 8 60–71. 10.1159/000487079 29606955 PMC5869579

[B121] McLean C. A. Cherny R. A. Fraser F. W. Fuller S. J. Smith M. J. Beyreuther K. (1999). Soluble pool of abeta Amyloid as a determinant of severity of neurodegeneration in Alzheimer’s disease. *Ann. Neurol.* 46 860–866. 10.1002/1531-8249(199912)46:6<860::aid-ana8>3.0.co;2-m 10589538

[B122] Min S. W. Cho S. H. Zhou Y. Schroeder S. Haroutunian V. Seeley W. W. (2010). Acetylation of tau inhibits its degradation and contributes to tauopathy. *Neuron* 67 953–966. 10.1016/j.neuron.2010.08.044 20869593 PMC3035103

[B123] Multhaup G. Huber O. Buée L. Galas M. C. (2015). Amyloid precursor protein (APP) metabolites APP intracellular fragment (AICD), Aβ42, and Tau in nuclear roles. *J. Biol. Chem.* 290 23515–23522. 10.1074/jbc.R115.677211 26296890 PMC4583011

[B124] Nazir M. A. (2017). Prevalence of periodontal disease, its association with systemic diseases and prevention. *Int. J. Health Sci. (Qassim)* 11 72–80. 28539867 PMC5426403

[B125] Neal M. Richardson J. R. (2018). Epigenetic regulation of astrocyte function in neuroinflammation and neurodegeneration. *Biochim. Biophys. Acta Mol. Basis Dis.* 1864 432–443. 10.1016/j.bbadis.2017.11.004 29113750 PMC5743548

[B126] Neely A. L. Holford T. R. Loe H. Anerud A. Boysen H. (2005). The natural history of periodontal disease in humans: risk factors for tooth loss in caries-free subjects receiving no oral health care. *J. Clin. Periodontol.* 32 984–893. 10.1111/j.1600-051X.2005.00797.x 16104963

[B127] Nilsberth C. Westlind-Danielsson A. Eckman C. B. Condron M. M. Axelman K. Forsell C. (2001). The ‘Arctic’ APP mutation (E693G) causes Alzheimer’s disease by enhanced Abeta protofibril formation. *Nat. Neurosci.* 4 887–893. 10.1038/nn0901-887 11528419

[B128] Noble J. M. Scarmeas N. Papapanou P. N. (2013). Poor oral health as a chronic, potentially modifiable dementia risk factor: review of the literature. *Curr. Neurol. Neurosci. Rep.* 13:384. 10.1007/s11910-013-0384-x 23963608 PMC6526728

[B129] Noble W. Hanger D. P. Miller C. C. Lovestone S. (2013). The importance of tau phosphorylation for neurodegenerative diseases. *Front. Neurol.* 4:83. 10.3389/fneur.2013.00083 23847585 PMC3696910

[B130] O’Brien R. J. Wong P. C. (2011). Amyloid precursor protein processing and Alzheimer’s disease. *Annu. Rev. Neurosci.* 34 185–204. 10.1146/annurev-neuro-061010-113613 21456963 PMC3174086

[B131] Osborn L. M. Kamphuis W. Wadman W. J. Hol E. M. (2016). Astrogliosis: an integral player in the pathogenesis of Alzheimer’s disease. *Prog. Neurobiol.* 144 121–141. 10.1016/j.pneurobio.2016.01.001 26797041

[B132] Passer B. Pellegrini L. Russo C. Siegel R. M. Lenardo M. J. Schettini G. (2000). Generation of an apoptotic intracellular peptide by γ-secretase cleavage of Alzheimer’s amyloid β protein precursor. *J. Alzheimers Dis.* 2 289–301. 10.3233/jad-2000-23-408 12214090

[B133] Pearson H. A. Peers C. (2006). Physiological roles for amyloid beta peptides. *J. Physiol.* 575(Pt 1) 5–10. 10.1113/jphysiol.2006.111203 16809372 PMC1819417

[B134] Perry V. H. Cunningham C. Holmes C. (2007). Systemic infections and inflammation affect chronic neurodegeneration. *Nat. Rev. Immunol.* 7 161–167. 10.1038/nri2015 17220915

[B135] Phillips E. C. Croft C. L. Kurbatskaya K. O’Neill M. J. Hutton M. L. Hanger D. P. (2014). Astrocytes and neuroinflammation in Alzheimer’s disease. *Biochem. Soc. Trans.* 42 1321–1325. 10.1042/BST20140155 25233410

[B136] Piccio L. Deming Y. Del-Águila J. L. Ghezzi L. Holtzman D. M. Fagan A. M. (2016). Cerebrospinal fluid soluble TREM2 is higher in Alzheimer disease and associated with mutation status. *Acta Neuropathol.* 131 925–933. 10.1007/s00401-016-1533-5 26754641 PMC4867123

[B137] Pinheiro L. Faustino C. (2019). Therapeutic strategies targeting amyloid-β in Alzheimer’s disease. *Curr. Alzheimer Res.* 16 418–452. 10.2174/1567205016666190321163438 30907320

[B138] Polvikoski T. Sulkava R. Haltia M. Kainulainen K. Vuorio A. Verkkoniemi A. (1995). Apolipoprotein E, dementia, and cortical deposition of beta-amyloid protein. *N. Engl. J. Med.* 333 1242–1247. 7566000 10.1056/NEJM199511093331902

[B139] Poole S. Singhrao S. K. Chukkapalli S. Rivera M. Velsko I. Kesavalu L. (2015). Active invasion of *Porphyromonas gingivalis* and infection-induced complement activation in ApoE–/– mice brains. *J. Alzheimers Dis.* 43 67–80. 10.3233/JAD-140315 25061055

[B140] Poole S. Singhrao S. K. Kesavalu L. Curtis M. A. Crean S. (2013). Determining the presence of periodontopathic virulence factors in short-term postmortem Alzheimer’s disease brain tissue. *J. Alzheimers. Dis.* 36 665–677. 10.3233/JAD-121918 23666172

[B141] Potempa J. Pike R. Travis J. (1995). The multiple forms of trypsin-like activity present in various strains of *Porphyromonas gingivalis* are due to the presence of either Arg-gingipain or Lys-gingipain. *Infect. Immun.* 63 1176–1182. 10.1128/iai.63.4.1176-1182.1995 7890369 PMC173131

[B142] Potempa J. Pike R. Travis J. (1997). Titration and mapping of the active site of cysteine proteinases from Porphyromonas gingivalis (gingipains) using peptidyl chloromethanes. *Biol. Chem.* 378 223–230. 9165075 10.1515/bchm.1997.378.3-4.223

[B143] Prince J. A. Zetterberg H. Andreasen N. Marcusson J. Blennow K. (2004). APOE epsilon4 allele is associated with reduced cerebrospinal fluid levels of Abeta42. *Neurology* 62 2116–2118. 10.1212/01.wnl.0000128088.08695.05 15184629

[B144] Ranjan R. Abhinay A. Mishra M. (2018). Can oral microbial infections be a risk factor for neurodegeneration? A review of the literature. *Neurol. India* 66 344–351. 10.4103/0028-3886.227315 29547153

[B145] Rodriguez G. A. Tai L. M. LaDu M. J. Rebeck G. W. (2014). Human APOE4 increases microglia reactivity at Aβ plaques in a mouse model of Aβ deposition. *J. Neuroinflamm.* 11:111. 10.1186/1742-2094-11-111 24948358 PMC4077554

[B146] Rolim T. S. Fabri G. M. Nitrini R. Anghinah R. Teixeira M. J. Siqueira J. T. (2014). Evaluation of patients with Alzheimer’s disease before and after dental treatment. *Arq. Neuropsiquiatr.* 72 919–924. 10.1590/0004-282X20140140 25517641

[B147] Roussos P. Katsel P. Fam P. Tan W. Purohit D. P. Haroutunian V. (2015). The triggering receptor expressed on myeloid cells 2 (TREM2) is associated with enhanced inflammation, neuropathological lesions and increased risk for Alzheimer’s dementia. *Alzheimers Dement.* 11 1163–1170. 10.1016/j.jalz.2014.10.013 25499537 PMC4461564

[B148] Scheltens P. Blennow K. Breteler M. M. de Strooper B. Frisoni G. B. Salloway S. (2016). Alzheimer’s disease. *Lancet* 388 505–517. 10.1016/S0140-6736(15)01124-1 26921134

[B149] Schmechel D. E. Saunders A. M. Strittmatter W. J. Crain B. J. Hulette C. M. Joo S. H. (1993). Increased amyloid beta-peptide deposition in cerebral cortex as a consequence of apolipoprotein E genotype in late-onset Alzheimer disease. *Proc. Natl. Acad. Sci. U.S.A.* 90 9649–9653. 10.1073/pnas.90.20.9649 8415756 PMC47627

[B150] Schöll M. Wall A. Thordardottir S. Ferreira D. Bogdanovic N. Långström B. (2012). Low PiB PET retention in presence of pathologic CSF biomarkers in Arctic APP mutation carriers. *Neurology* 79 229–236. 10.1212/WNL.0b013e31825fdf18 22700814

[B151] Sengupta A. Kabat J. Novak M. Wu Q. Grundke-Iqbal I. Iqbal K. (1998). Phosphorylation of tau at both Thr 231 and Ser 262 is required for maximal inhibition of its binding to microtubules. *Arch. Biochem. Biophys.* 357 299–309. 10.1006/abbi.1998.0813 9735171

[B152] Serpell L. C. (2000). Alzheimer’s amyloid fibrils: structure and assembly. *Biochim. Biophys. Acta* 1502 16–30. 10899428 10.1016/s0925-4439(00)00029-6

[B153] Sharma A. (2010). Virulence mechanisms of *Tannerella forsythia*. *Periodontology* 54 106–116. 10.1111/j.1600-0757.2009.00332.x 20712636 PMC2934765

[B154] Sheng J. G. Bora S. H. Xu G. Borchelt D. R. Price D. L. Koliatsos V. E. (2003). Lipopolysaccharide-induced-neuroinflammation increases intracellular accumulation of amyloid precursor protein and amyloid beta peptide in APPswe transgenic mice. *Neurobiol. Dis.* 14 133–145. 10.1016/s0969-9961(03)00069-x 13678674

[B155] Shenker B. J. Besack D. McKay T. Pankoski L. Zekavat A. Demuth D. R. (2005). Induction of cell cycle arrest in lymphocytes by *Actinobacillus actinomycetemcomitans* cytolethal distending toxin requires three subunits for maximum activity. *J. Immunol.* 174 2228–2234. 10.4049/jimmunol.174.4.2228 15699156

[B156] Shi Y. Yamada K. Liddelow S. A. Smith S. T. Zhao L. Luo W. (2017). ApoE4 markedly exacerbates tau-mediated neurodegeneration in a mouse model of tauopathy. *Nature* 549 523–527. 10.1038/nature24016 28959956 PMC5641217

[B157] Shimada H. Ataka S. Tomiyama T. Takechi H. Mori H. Miki T. (2011). Clinical course of patients with familial early-onset Alzheimer’s disease potentially lacking senile plaques bearing the E693Δ mutation in amyloid precursor protein. *Dement. Geriatr. Cogn. Disord.* 32 45–54. 10.1159/000330017 21846988

[B158] Sims R. van der Lee S. J. Naj A. C. Bellenguez C. Badarinarayan N. Jakobsdottir J. (2017). Rare coding variants in PLCG2, ABI3, and TREM2 implicate microglial-mediated innate immunity in Alzheimer’s disease. *Nat. Genet.* 49 1373–1384. 10.1038/ng.3916 28714976 PMC5669039

[B159] Singhrao S. K. Chukkapalli S. Poole S. Velsko I. Crean S. J. Kesavalu L. (2017). Chronic *Porphyromonas gingivalis* infection accelerates the occurrence of age-related granules in ApoE-/- mice brains. *J. Oral Microbiol.* 9:1270602. 10.1080/20002297.2016.1270602 28326151 PMC5328363

[B160] Singhrao S. K. Harding A. Poole S. Kesavalu L. Crean S. (2015). Porphyromonas gingivalis Periodontal Infection and Its Putative Links with Alzheimer’s Disease. *Mediat. Inflamm.* 2015:137357. 10.1155/2015/137357 26063967 PMC4430664

[B161] Sochalska M. Potempa J. (2017). Manipulation of Neutrophils by *Porphyromonas gingivalis* in the Development of Periodontitis. *Front. Cell Infect. Microbiol.* 7:197. 10.3389/fcimb.2017.00197 28589098 PMC5440471

[B162] Socransky S. S. Haffajee A. D. Cugini M. A. Smith C. Kent R. L. Jr. (1998). Microbial complexes in subgingival plaque. *J. Clin. Periodontol.* 25 134–144. 10.1111/j.1600-051x.1998.tb02419.x 9495612

[B163] Sofroniew M. V. Vinters H. V. (2010). Astrocytes: biology and pathology. *Acta Neuropathol.* 119 7–35. 10.1007/s00401-009-0619-8 20012068 PMC2799634

[B164] Solito E. Sastre M. (2012). Microglia function in Alzheimer’s disease. *Front. Pharmacol.* 3:14. 10.3389/fphar.2012.00014 22363284 PMC3277080

[B165] Song W. Hooli B. Mullin K. Jin S. C. Cella M. Ulland T. K. (2017). Alzheimer’s disease-associated TREM2 variants exhibit either decreased or increased ligand-dependent activation. *Alzheimers Dement.* 13 381–387. 10.1016/j.jalz.2016.07.004 27520774 PMC5299056

[B166] Sosa-Ortiz A. L. Acosta-Castillo I. Prince M. J. (2012). Epidemiology of dementias and Alzheimer’s disease. *Arch. Med Res.* 43 600–608. 10.1016/j.arcmed.2012.11.003 23159715

[B167] Sparks S. P. Steffen M. J. Smith C. Jicha G. Ebersole J. L. Abner E. (2012). Serum antibodies to periodontal pathogens are a risk factor for Alzheimer’s disease. *Alzheimers Dement.* 8 196–203. 10.1016/j.jalz.2011.04.006 22546352 PMC3712346

[B168] Sperling R. A. Aisen P. S. Beckett L. A. Bennett D. A. Craft S. Fagan A. M. (2011). Toward defining the preclinical stages of Alzheimer’s disease: recommendations from the National Institute on Aging-Alzheimer’s Association workgroups on diagnostic guidelines for Alzheimer’s disease. *Alzheimers Dement.* 7 280–292. 21514248 10.1016/j.jalz.2011.03.003 PMC3220946

[B169] Stalder M. Deller T. Staufenbiel M. Jucker M. (2001). 3D-Reconstruction of microglia and amyloid in APP23 transgenic mice: no evidence of intracellular amyloid. *Neurobiol. Aging* 22 427–434. 10.1016/s0197-4580(01)00209-3 11378249

[B170] Steardo L. Jr. Bronzuoli M. R. Iacomino A. Esposito G. Steardo L. Scuderi C. (2015). Does neuroinflammation turn on the flame in Alzheimer’s disease? Focus on astrocytes. *Front. Neurosci.* 9:259. 10.3389/fnins.2015.00259 26283900 PMC4518161

[B171] Struhl G. Greenwald I. (1999). Presenilin is required for activity and nuclear access of Notch in Drosophila. *Nature* 398 522–525. 10.1038/19091 10206646

[B172] Sudhakara P. Gupta A. Bhardwaj A. Wilson A. (2018). Oral dysbiotic communities and their implications in systemic diseases. *Dent. J. (Basel)* 6:E10. 10.3390/dj6020010 29659479 PMC6023521

[B173] Sung C. E. Huang R. Y. Cheng W. C. Kao T. W. Chen W. L. (2019). Association between periodontitis and cognitive impairment: analysis of national health and nutrition examination survey (NHANES) III. *J. Clin. Periodontol.* 46 790–798. 10.1111/jcpe.13155 31152592

[B174] Szekely C. A. Breitner J. C. Fitzpatrick A. L. Rea T. D. Psaty B. M. Kuller L. H. (2008). NSAID use and dementia risk in the cardiovascular health study: role of APOE and NSAID type. *Neurology* 70 17–24. 10.1212/01.wnl.0000284596.95156.48 18003940 PMC2877629

[B175] Tada A. Senpuku H. Motozawa Y. Yoshihara A. Hanada N. Tanzawa H. (2006). Association between commensal bacteria and opportunistic pathogens in the dental plaque of elderly individuals. *Clin. Microbiol. Infect.* 12 776–781. 10.1111/j.1469-0691.2006.01497.x 16842573

[B176] Tanzi R. E. (2015). TREM2 and risk of Alzheimer’s disease–friend or foe? *N. Engl. J. Med.* 372 2564–2565. 10.1056/NEJMcibr1503954 26107057

[B177] Tejera D. Mercan D. Sanchez-Caro J. M. Hanan M. Greenberg D. Soreq H. (2019). Systemic inflammation impairs microglial Aβ clearance through NLRP3 inflammasome. *EMBO J.* 38:e101064. 10.15252/embj.2018101064 31359456 PMC6717897

[B178] Tomiyama T. Matsuyama S. Iso H. Umeda T. Takuma H. Ohnishi K. (2010). A mouse model of amyloid beta oligomers: their contribution to synaptic alteration, abnormal tau phosphorylation, glial activation, and neuronal loss in vivo. *J. Neurosci.* 30 4845–4856. 10.1523/JNEUROSCI.5825-09.2010 20371804 PMC6632783

[B179] Tzeng N. S. Chung C. H. Lin F. H. Chiang C. P. Yeh C. B. Huang S. Y. (2018). Anti-herpetic medications and reduced risk of dementia in patients with herpes simplex virus infections-a nationwide, population-based cohort study in Taiwan. *Neurotherapeutics* 15 417–429. 10.1007/s13311-018-0611-x 29488144 PMC5935641

[B180] Van de Haar H. J. Burgmans S. Jansen J. F. van Osch M. J. van Buchem M. A. Muller M. (2016). Blood-brain barrier leakage in patients with early Alzheimer disease. *Radiology* 281 527–535. 10.1148/radiol.2016152244 27243267

[B181] Verkhratsky A. Olabarria M. Noristani H. N. Yeh C. Y. Rodriguez J. J. (2010). Astrocytes in Alzheimer’s disease. *Neurotherapeutics* 7 399–412. 10.1016/j.nurt.2010.05.017 20880504 PMC5084302

[B182] Walsh D. M. Selkoe D. J. (2007). Aβ oligomers: a decade of discovery. *J. Neurochem.* 101 1172–1184. 10.1111/j.1471-4159.2006.04426.x 17286590

[B183] Wang Y. Cella M. Mallinson K. Ulrich J. D. Young K. L. Robinette M. L. (2015). TREM2 lipid sensing sustains the microglial response in an Alzheimer’s disease model. *Cell* 160 1061–1071. 10.1016/j.cell.2015.01.049 25728668 PMC4477963

[B184] Weidemann A. Eggert S. Reinhard F. B. M. Vogel M. Paliga K. Baier G. (2002). A novel epsilon-cleavage within the transmembrane domain of the alzheimer amyloid precursor protein demonstrates homology with notch processing. *Biochemistry* 41 2825–2835. 10.1021/bi015794o 11851430

[B185] Weingarten M. D. Lockwood A. H. Hwo S. Y. Kirschner M. W. (1975). A protein factor essential for microtubule assembly. *Proc. Natl. Acad. Sci. U.S.A.* 72 1858–1862. 10.1073/pnas.72.5.1858 1057175 PMC432646

[B186] Wolf S. A. Boddeke H. W. Kettenmann H. (2017). Microglia in physiology and disease. *Annu. Rev. Physiol.* 79 619–643. 10.1146/annurev-physiol-022516-034406 27959620

[B187] Wolfe M. S. Xia W. Moore C. L. Leatherwood D. D. Ostaszewski B. Rahmati T. (1999). Peptidomimetic probes and molecular modeling suggest that Alzheimer’s gamma-secretase is an intramembrane-cleaving aspartyl protease. *Biochemistry* 38 4720–4727. 10.1021/bi982562p 10200159

[B188] Wozniak M. A. Mee A. P. Itzhaki R. F. (2009). Herpes simplex virus type 1 DNA is located within Alzheimer’s disease amyloid plaques. *J. Pathol.* 217 131–138. 10.1002/path.2449 18973185

[B189] Yeh F. L. Wang Y. Tom I. Gonzalez L. C. Sheng M. (2016). TREM2 binds to apolipoproteins, including APOE and CLU/APOJ, and thereby facilitates uptake of amyloid-Beta by microglia. *Neuron* 91 328–340. 10.1016/j.neuron.2016.06.015 27477018

[B190] Yilmaz O. (2008). The chronicles of Porphyromonas gingivalis: the microbium, the human oral epithelium and their interplay. *Microbiology* 154(Pt 10), 2897–2903. 10.1099/mic.0.2008/021220-0 18832296 PMC2639765

[B191] Yoo J. J. Yoon J. H. Kang M. J. Kim M. Oh N. (2019). The effect of missing teeth on dementia in older people: a nationwide population-based cohort study in South Korea. *BMC Oral Health* 19:61. 10.1186/s12903-019-0750-4 31023356 PMC6485168

[B192] Zhan X. Stamova B. Jin L. W. DeCarli C. Phinney B. Sharp F. R. (2016). Gram-negative bacterial molecules associate with Alzheimer disease pathology. *Neurology* 87 2324–2332. 10.1212/wnl.0000000000003391 27784770 PMC5135029

[B193] Zhan X. Stamova B. Sharp F. R. (2018). Lipopolysaccharide associates with amyloid plaques, neurons and oligodendrocytes in Alzheimer’s disease brain: a review. *Front. Aging Neurosci.* 10:42. 10.3389/fnagi.2018.00042 29520228 PMC5827158

[B194] Zhao Y. Cong L. Jaber V. Lukiw W. J. (2017). Microbiome-derived lipopolysaccharide enriched in the perinuclear region of Alzheimer’s disease brain. *Front. Immunol.* 8:1064. 10.3389/fimmu.2017.01064 28928740 PMC5591429

[B195] Zuroff L. Daley D. Black K. L. Koronyo-Hamaoui M. (2017). Clearance of cerebral Aβ in Alzheimer’s disease: reassessing the role of microglia and monocytes. *Cell Mol. Life Sci.* 74 2167–2201. 10.1007/s00018-017-2463-7 28197669 PMC5425508

